# Complications Following Mandibular Cheek Tooth Extraction in 20 Horses

**DOI:** 10.3389/fvets.2020.00504

**Published:** 2020-08-13

**Authors:** Hauke Gergeleit, Astrid Bienert-Zeit

**Affiliations:** Clinic for Horses, University of Veterinary Medicine Hannover, Hannover, Germany

**Keywords:** equine, exodontia, fistula, tooth sectioning, sequestrum

## Abstract

The objectives of this retrospective study were to describe the prevalence and characteristics of post-operative complications that occur following equine mandibular cheek tooth extractions and to assess for possible associated risk factors. Clinically significant post-extraction complications necessitating repeat referral developed following 20/302 (6.6%) mandibular cheek tooth extractions. Horses developing complications were younger than the overall population having mandibular cheek teeth extractions and the most commonly affected teeth were the Triadan 07 s and 09 s. Alveolar sequestration was the most prevalent complication, occurring in 18/20 horses (90%), with the complete alveolus sequestering in some cases. Post-extraction mandibular fistula formation occurred in 5/20 cases (25%) and mandibular abscessation in 4/20 cases (20%). All cases were successfully treated, including sequestrectomy, and wound debridement with some cases taking up to 5 months for resolution. Anatomical features of the equine mandibular alveoli and bone appears to make them more prone to develop extensive sequestration compared to published complications on maxillary alveolar bone. This requires good pre-operative examination including diagnostic imaging to identify cases of higher risk and thorough risk disclosure toward horse owners as well as owners' compliance.

## Introduction

Dental extractions are standard procedures in horses with diseased cheek teeth, especially apical infections, but they are associated with a higher prevalence of complications compared to other commonly performed surgical procedures (1–3). However, the development of newer instrumentation for less invasive techniques, improvements in training and advancements in sedation and analgesia have negated the need for standard dental repulsions under general anesthesia and have made dental surgeries less debilitating for horses (4).

Reported post-extraction complication rates differ remarkably among studies. The highest complication rates are reported for repulsion of cheek teeth under general anesthesia with up to 80% post-operative complications (2, 5) whereas standing oral extractions have considerably lower complication rates of 4–20% (2, 6, 7) making this the preferred method whenever possible.

Despite the above advances, complications still occur with equine cheek tooth extractions with an apparently higher prevalence with mandibular (18.1%) than maxillary (9.7%) cheek teeth extractions (3).

The aims of this study were to describe clinical and demographic findings of horses that developed clinically significant complications following mandibular cheek tooth extraction, and to describe possible risk factors for their development. This knowledge will hopefully allow objective information on the risks of mandibular cheek teeth extraction to be disseminated.

## Materials and Methods

Clinical records of all equine cheek tooth exodontias performed between January 2014 to December 2019 at the Equine Clinic, University of Veterinary Medicine Hannover were examined. Data obtained included demographic and case details, Triadan position of affected teeth, number of teeth extracted and the extraction technique. Diseased cheek teeth that were readily extracted (either by hand or within 15 min with use of forceps) were not included in these data.

Maxillary or mandibular cheek teeth extraction records were separated, and mandibular extraction records were reviewed for the presence of post-operative complications. From these records, case details and clinical findings from the initial general and oral examinations, radiographic findings, diagnosis, surgical procedure and post-operative findings were thoroughly examined and tabulated. If teeth could not be extracted *per os* with forceps, the number and types of additional exodontia procedures were recorded. Where teeth were not extracted intact, post-operative radiographs were used to confirm complete cheek tooth extraction.

A complication associated with mandibular cheek tooth removal was defined as a case that: required additional post-extraction treatments in addition to standard alveolar swab changes; had delayed healing (>8 weeks for complete gingival epithelization); significant increase in treatment costs and/or moderate to severe post-extraction discomfort. Minor complications including reduced food intake or mild swellings that resolved within 2–3 days following extraction that were considered clinically insignificant and did not cause delayed alveolar healing were not included in this study.

### Standard Post-operative Management

Horses received flunixin meglumine (Flunidol® CP-Pharma Handelsgesellschaft mbH, Burgdorf, Germany) twice daily 1.1 mg/kg bwt for 3 days followed by 0.55 mg/kg for two days. Horses received systemic antibiotics (trimethoprim sulfadiazine: Synutrim® Vétoquinol GmbH, Ismaning, Germany) 30 mg/kg bwt twice daily pre-operatively only if they had a pre-existing mandibular fistula, moderate to severe pain on mandibular palpation or during mastication or if an additional technique to oral extraction was required. In these horses, antibiotic treatment was started ~12 h before the operation and continued for 5–10 days following the extraction depending on the clinical course.

Following extraction, the alveoli were lavaged with water with mild pressure to remove debris and pus and a surgical swab impregnated with medical grade honey (Mielosan® CP-Pharma Handelsgesellschaft mbH, Burgdorf, Germany) was placed in the alveoli. The alveolus was examined oroscopically and by digital palpation, and the initial swab was replaced in our clinic, usually at 2 days post-surgery, and weekly afterwards by the referring veterinarian until there was almost complete alveolar healing.

Follow-up information on post-operative complications associated with the underlying disease or the extraction technique was obtained from the clinical records, the referring veterinarian or by informal telephone interviews with owners.

## Results

### Case Overview

**Figure 1:**
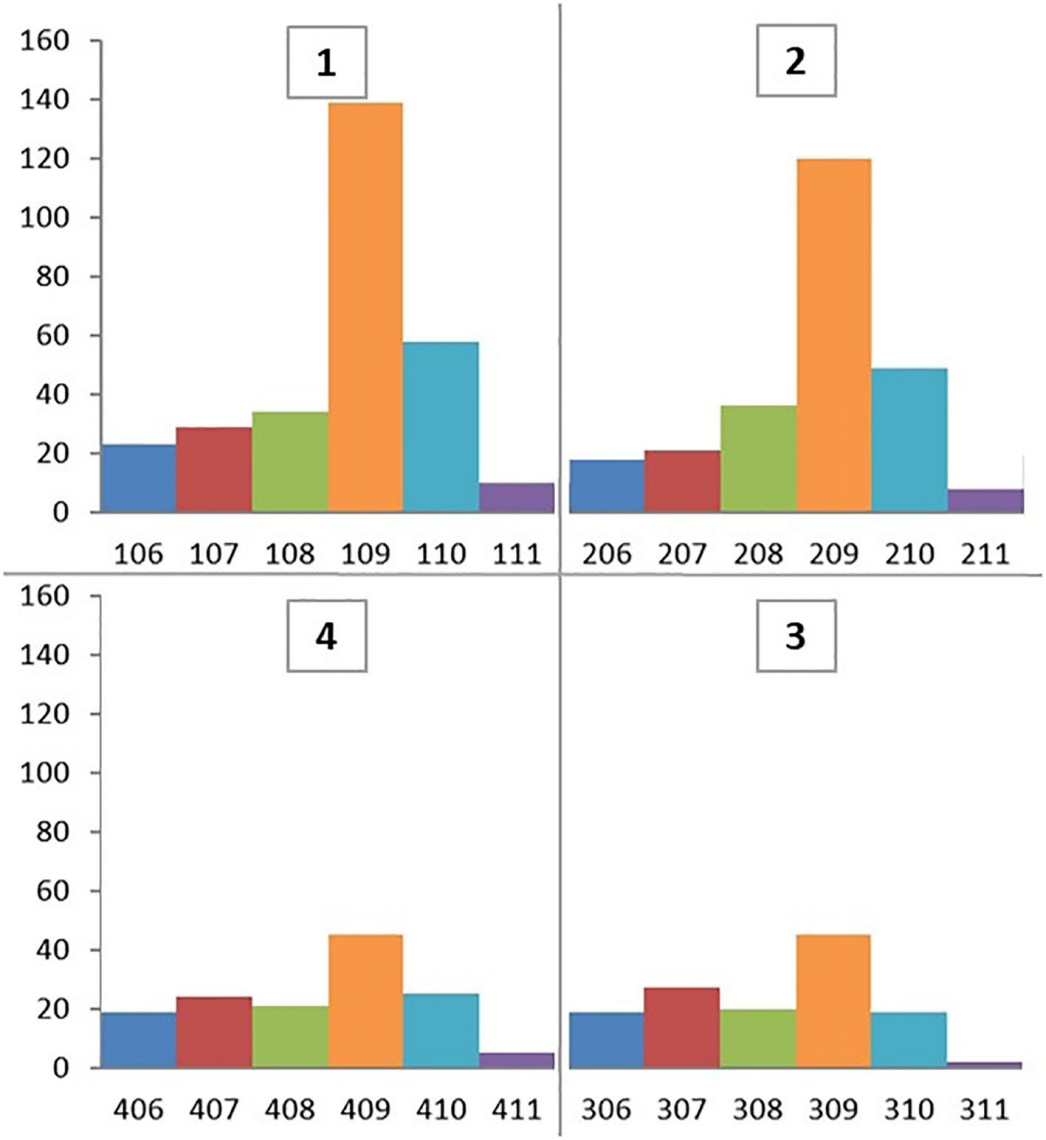
Triadan positions of 880 cheek teeth extracted between January 2014 and December 2019.

A total of 880 cheek teeth extractions including 578 (65.7%) maxillary and 302 (34.3%) mandibular teeth were performed in 561 horses, at 653 dental procedures (Figure 1). Patients included 48.9% females, 48.1% geldings, and 3% stallions of a mean of 13.3 ± 6.1 years old (range 3–29 years) with a similar age distribution for maxillary (mean 13.5 ± 5.9 years) and mandibular cheek teeth (mean 13 ± 6.7 years).

Twenty of the 302 (6.6%) extracted mandibular cheek teeth developed clinically significant post-operative complications necessitating re-referral back to this clinic (Table 1) and all required longer post- extraction treatment than usual. During this period, no horses were referred back to us because of maxillary alveolar sequestration. The prevalence of post-extraction complications was highest for mandibular Triadan 07 s (7/55 horses; 12.7%) and 09 s (7/101 horses; 6.9%). Horses developing complications were considerably younger (mean 7.3 ± 3.7 years; range 3–14 years) than all horses undergoing mandibular tooth extraction (mean 13 ± 6.7 years).

**Table 1:** Case details for horses with post-operative complications following mandibular cheek tooth removal.

**No**.	**Age (years)**	**External swelling^**†**^**	**Draining tract^**†**^**	**Triadan**	**Underlying disease**	**Tooth deformity**	**First surgery**	**Second surgery**	**Complications**
1	13	NO	NO	409	Fractured crown	YES	Sectioning	Buccotomy	Sequestrum
2	4	YES	NO	407	Pulpitis, Displacement	YES	Buccotomy	n.a.	Buccal abscess, Sequestrum
3	13	NO	NO	407	Fractured crown, Pulpitis	NO	Extraction	Extraction root fragment	Fistula, Sequestrum
4	4	NO	NO	410	Diastemata, Periodontal disease	NO	Extraction	n.a.	Sequestrum
5	4	NO	NO	309	Pulpitis	NO	Extraction	n.a.	Sequestrum
6	14	NO	NO	410-12	Displacement, Periodontal disease	NO	Extraction	n.a.	Sequestrum, Abscessation mandible
7	6	NO	NO	409	Diastemata, Displacement, Periodontal disease	YES	Extraction	n.a.	Sequestrum, Abscessation mandible
8	7	YES	NO	406	Periodontal disease after extraction of 405	NO	Extraction	n.a.	Sequestrum, Fistula
9	10	YES	YES	306	Periodontal disease after extraction of 305	NO	Extraction	n.a.	Fistula post extraction 305
10	3	YES	NO	406	Apical infection	YES	Extraction	n.a.	Sequestrum
11	6	NO	NO	306	Apical infection	NO	Extraction	n.a.	Sequestrum
12	4	YES	NO	307	Pulpitis, Apical infection	YES	Extraction	n.a.	Sequestrum
13	11	NO	NO	309	Fractured crown, Pulpitis	NO	Extraction	Sectioning, Screw-extraction	Sequestrum
14	8	YES	NO	307	Apical infection	NO	Extraction	n.a.	Sequestrum, Fistula
15	13	NO	NO	409	Fractured crown, Pulpitis	NO	Extraction, Sectioning	n.a.	Abscessation mandible
16	5	YES	YES	407	Apical infection	YES	Extraction, Sectioning, Repulsion	n.a.	Sequestrum
17	4	YES	YES	407	Apical infection	YES	Extraction	Repulsion	Sequestrum
18	7	YES	YES	307	Apical infection	YES	Extraction, Repulsion	n.a.	Sequestrum
19	10	YES	NO	309	Diastemata, Displacement, Periodontal disease	NO	Extraction	Extraction	Sequestrum, Abscessation mandible
20	13	NO	NO	409	Pulpitis	NO	Extraction	n.a.	Sequestrum, Fistula

† *On initial presentation; n.a., not applicable*.

### Initial Clinical Findings and Diagnostic Imaging Findings of 20 Cases Developing Complications

**Figure 2:**
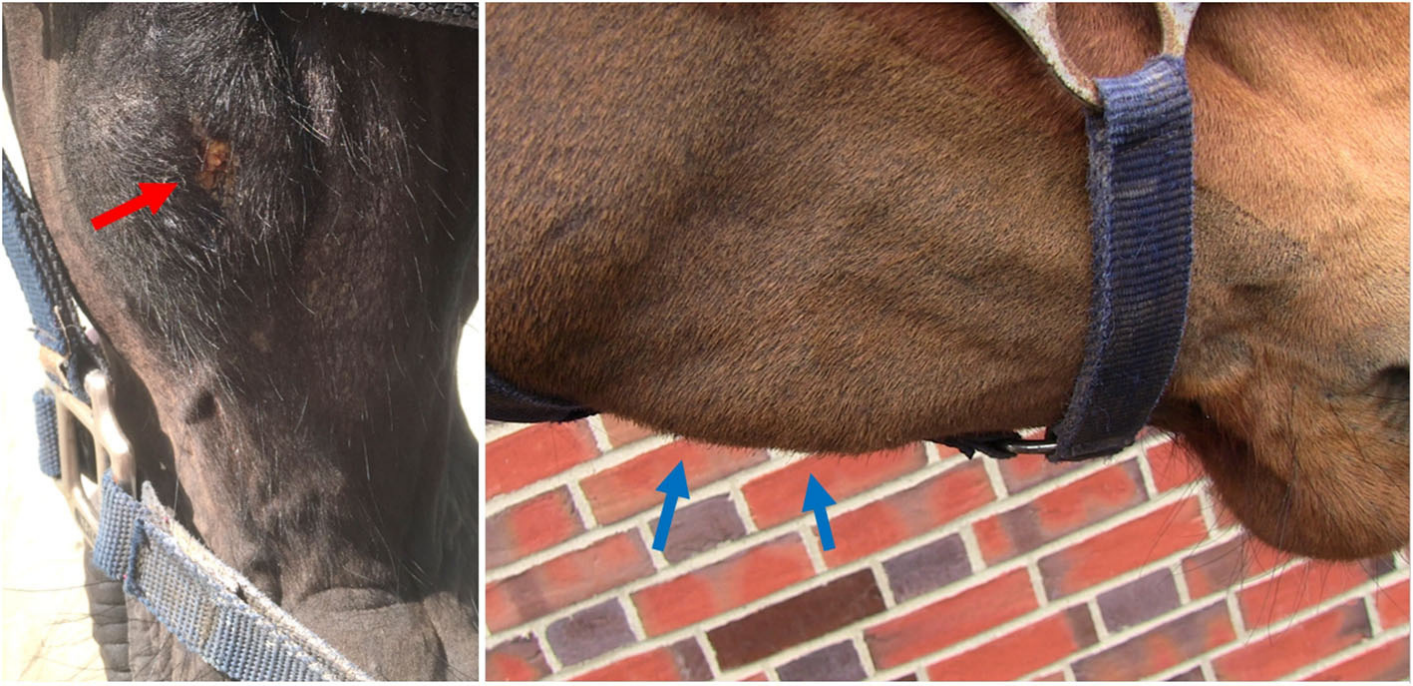
Swelling of the left mandible with a purulent external draining tract (left, red arrow, Horse No. 18). A firm swelling of the right mandible (right, blue arrows, Horse No. 16). Both horses had apical infections.

**Figure 3:**
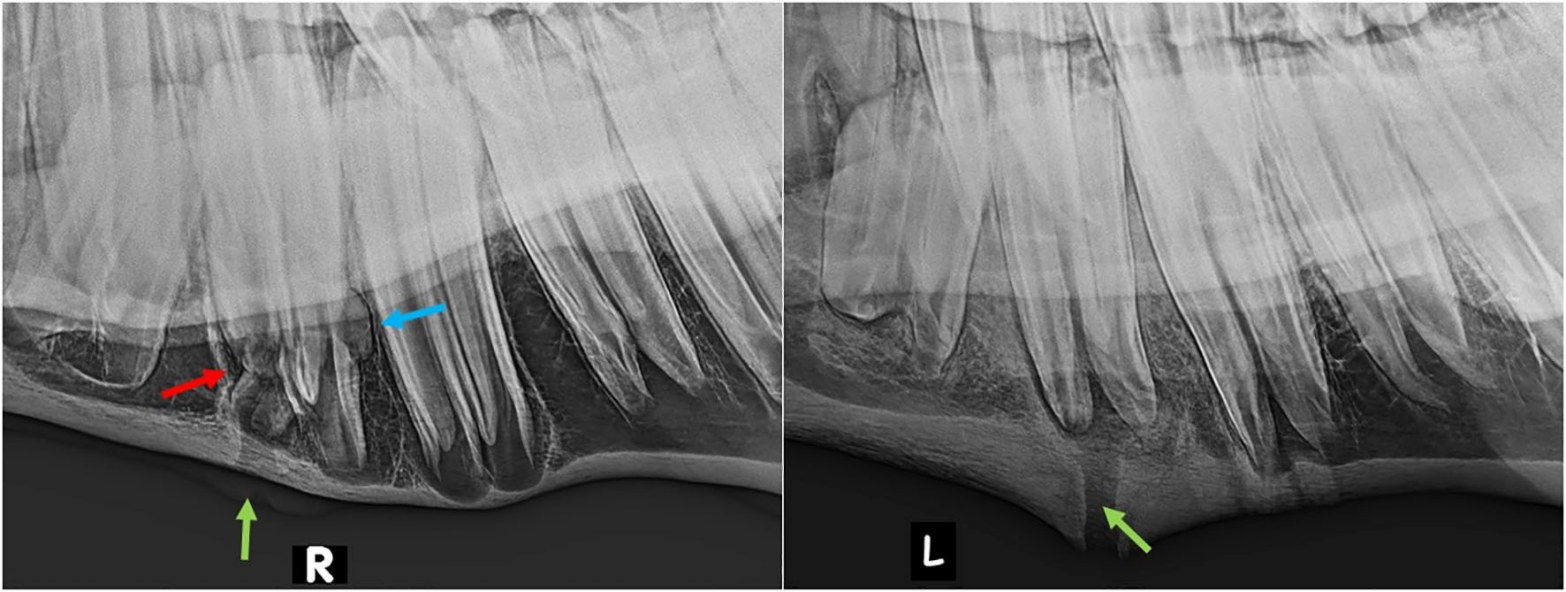
Lateral oblique radiographs of the mandibles of two horses with apical infection. Left (Horse No. 16): External swelling and fistula from the mesial aspect of the 407 (green arrow). The mesio-distal aspect is markedly malformed (red arrow) and there is also a radiopaque enlargement on the distal aspect of the tooth (blue arrow). Right (Horse No. 18): External swelling and fistula from the mesial root of the 307 (green arrow).

Ten of the 20 horses that developed complications had mandibular swellings with four of these also having an external draining tract (fistula) (Figure 2). Radiography with use of a probe showed the fistulas to be associated with Triadan 07 s apices (*n* = 3) and Triadan 06 (*n* = 1). Infection-related apical malformation and enlargement was found in eight horses (Figure 3).

The diagnosed dental problem necessitating exodontia in these 20 teeth was apical infection (*n* = 6), endodontic diseases (*n* = 8, including due partial fractures and pulpitis) and periodontal diseases (*n* = 6, including diastemata and tooth displacement).

Horses with apical infections were a mean of 5.5 (range 3–8) years old and had infected Triadan 07 s (*n* = 4) and 06 s (*n* = 2). Horses with endodontic and periodontal disease were a mean of 9 (range 4–14) years old with affected teeth in all Triadan positions, except Triadan 08.

### Surgical Techniques

**Figure 4:**
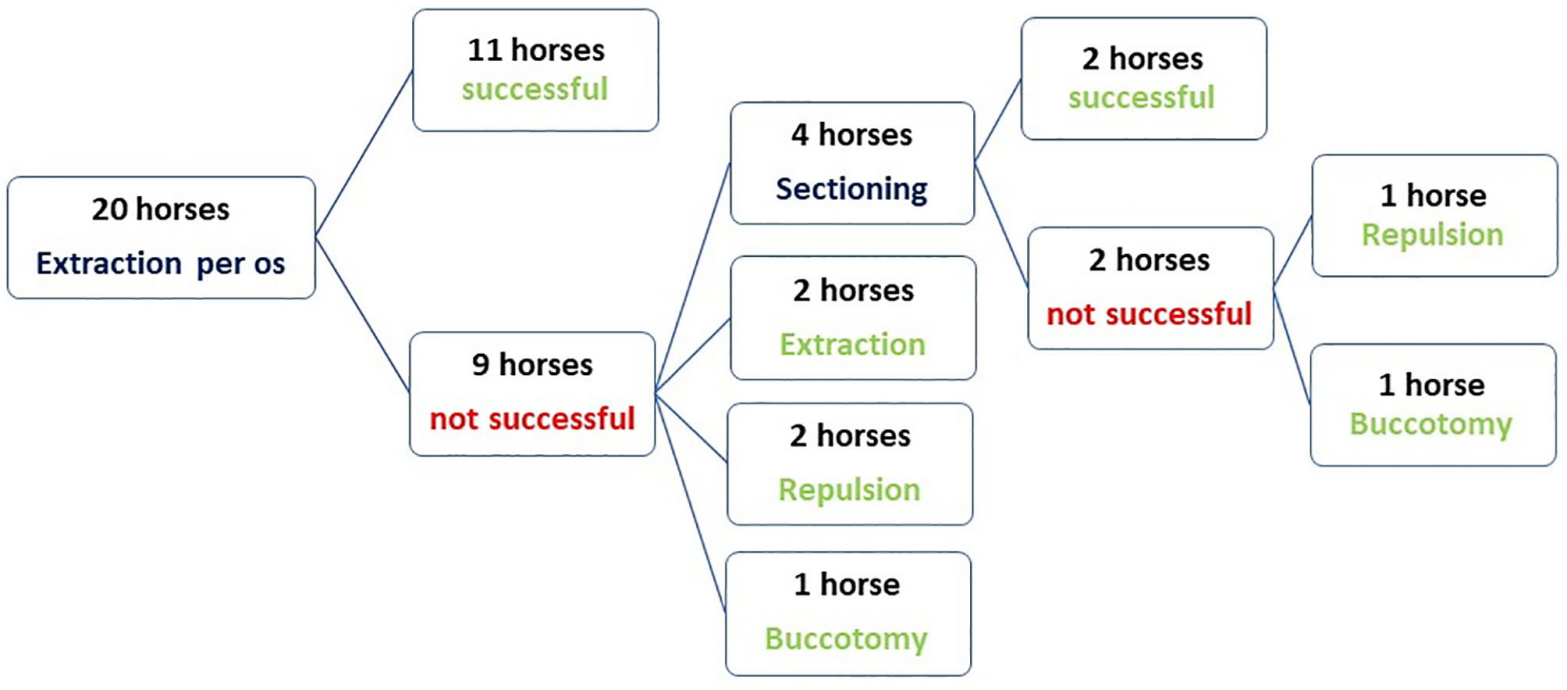
Flow chart showing the techniques used for exodontia of mandibular cheek teeth in 20 horses that later developed post-operative complications.

Oral extraction with forceps in deeply sedated horses, following a mandibular nerve block and local infiltrative anesthesia was initially attempted in all cases. This technique was successful at the initial surgery in 11/20 horses (55%) taking between 30 min and 4 h, including preparation time. However, additional techniques (described below) were required in 7/20 horses (35%) (Figure 4). In two other horses, the affected tooth was extracted per *os* at a second surgery.

**Figure 5:**
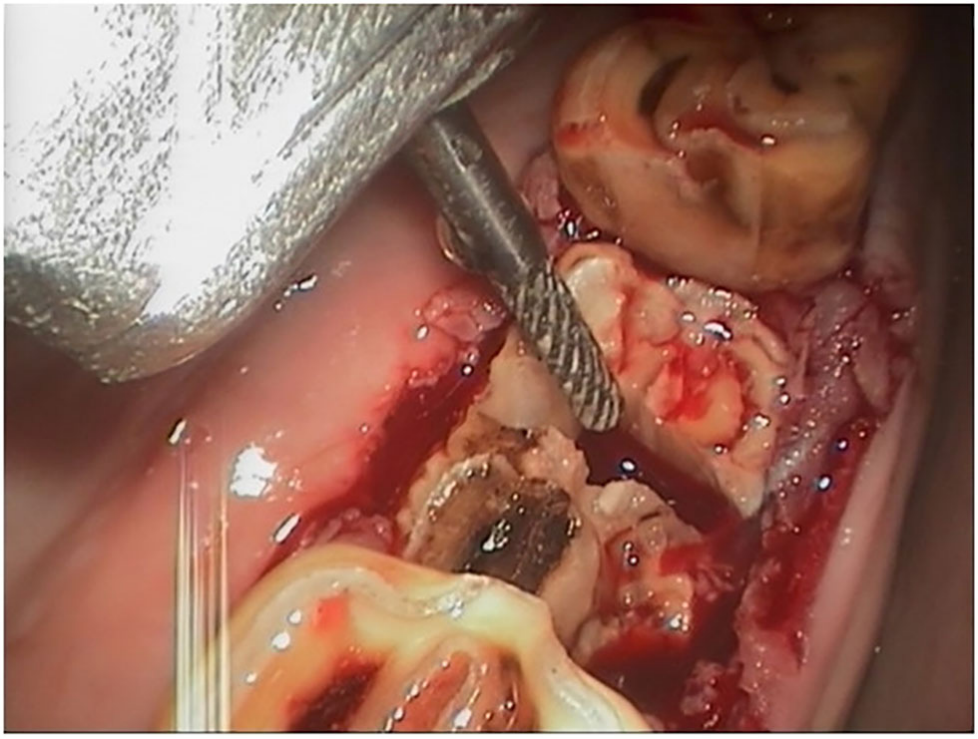
Intraoral sectioning of a 407 under endoscopic control after the crown fractured during oral extraction. The bur is used to section the tooth in a lingual to buccal (transverse) direction to allow separate extraction of the mesial and distal parts of the tooth (buccal is to the left).

Intraoral sectioning of the tooth under endoscopic guidance was performed during the first procedure in three horses and in one horse at a second procedure which allowed complete extraction in two of these four cases. Tooth sectioning was performed with a motorized instrument and a 3 mm diameter double cut carbide burs of different length. Teeth were sectioned on a lingual to buccal transverse direction between pulp horns 1 and 2 in order to create a gap and separate the mesial and distal part of the tooth (Figure 5). Intraoral water lavage was used to cool the instrument and the tooth during sectioning. In the two cases where sectioning failed the tooth was successfully repulsed via the preexisting fistula in one case and the other case had a minimally invasive buccotomy to successfully remove the remaining dental fragment. A minimally invasive buccotomy was successfully utilized during the first procedure in one of the remaining cases and a screw extraction during a second procedure in one other case. Overall, five horses required a second surgery 2–3 days following the first attempt to complete the exodontia (Figure 4).

### Post-operative Complications

**Figure 6:**
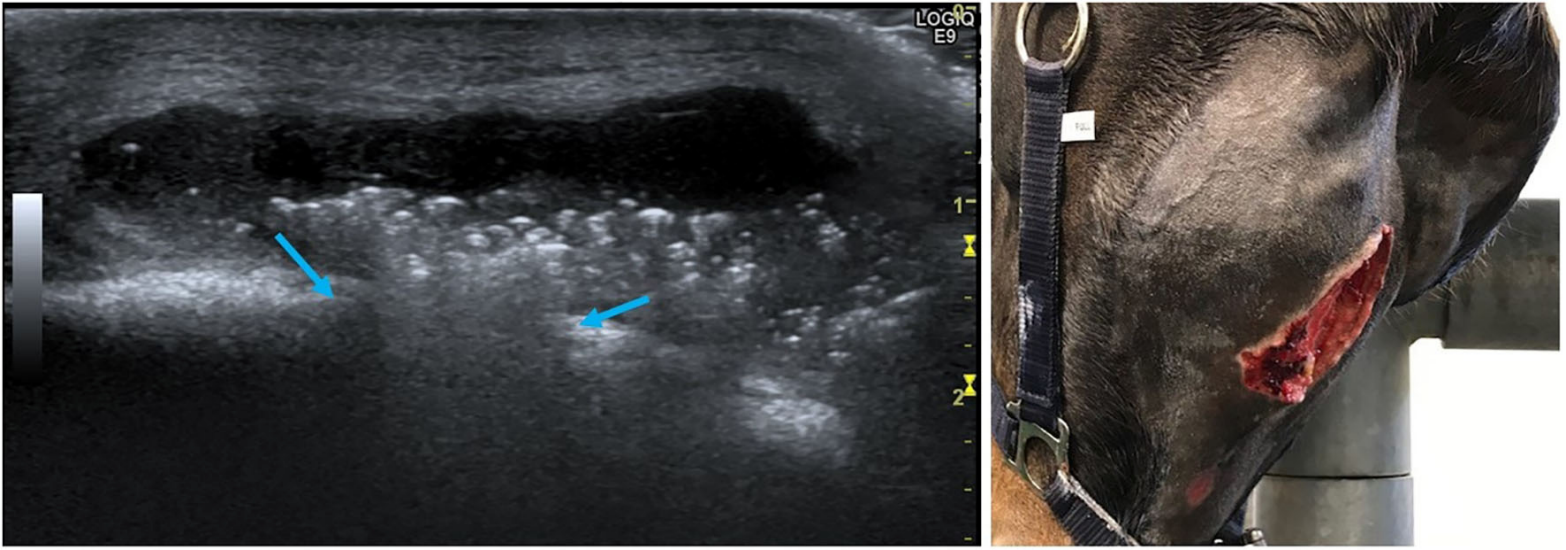
Left (Horse No. 14): Ultrasonographic image of mandibular abscess formation after extraction of a 307 due to apical infection. The bone contour is interrupted (blue arrows) and the abscess presents as an anechogenic fluid-filled cavity with heterogeneous hyperechogenic spots. The skin is still intact (upper side of the image is ventral). Right (Horse No. 20): Extensive skin sloughing has occurred 7 weeks after 309 extraction and mandibular abscess formation.

The most prevalent significant post-operative complication necessitating re-referral, occurring in 18/20 horses (90%) was alveolar bone sequestration with consequent delayed alveolar healing. The overall prevalence of clinically significant alveolar sequestration following mandibular extractions was 6% (18/302). Other complications included post-operative mandibular abscess and fistula formation (*n* = 7). Abscess formation at the buccotomy site (*n* = 1) developed within 2 days of surgery. Following drainage and routine wound lavage, it healed completely by secondary intention within 3 weeks. Mandibular abscesses drained spontaneously (*n* = 2) two and seven weeks following the tooth extraction or were incised under ultrasonographic guidance (*n* = 2) 1 week and 4 months, respectively, following the tooth extraction (Figure 6).

Alveolar healing problems usually became clinically obvious 1–2 weeks post-extraction. These disorders were detected by the referring veterinarian during routine post-extraction examinations or by the owners recognizing enlarging and painful mandibular swellings and problems in these horses when eating (slow chewing, quidding, or inappetence). All 20 horses were referred back for treatment at this clinic, 2–8 weeks following their initial discharge.

**Figure 7:**
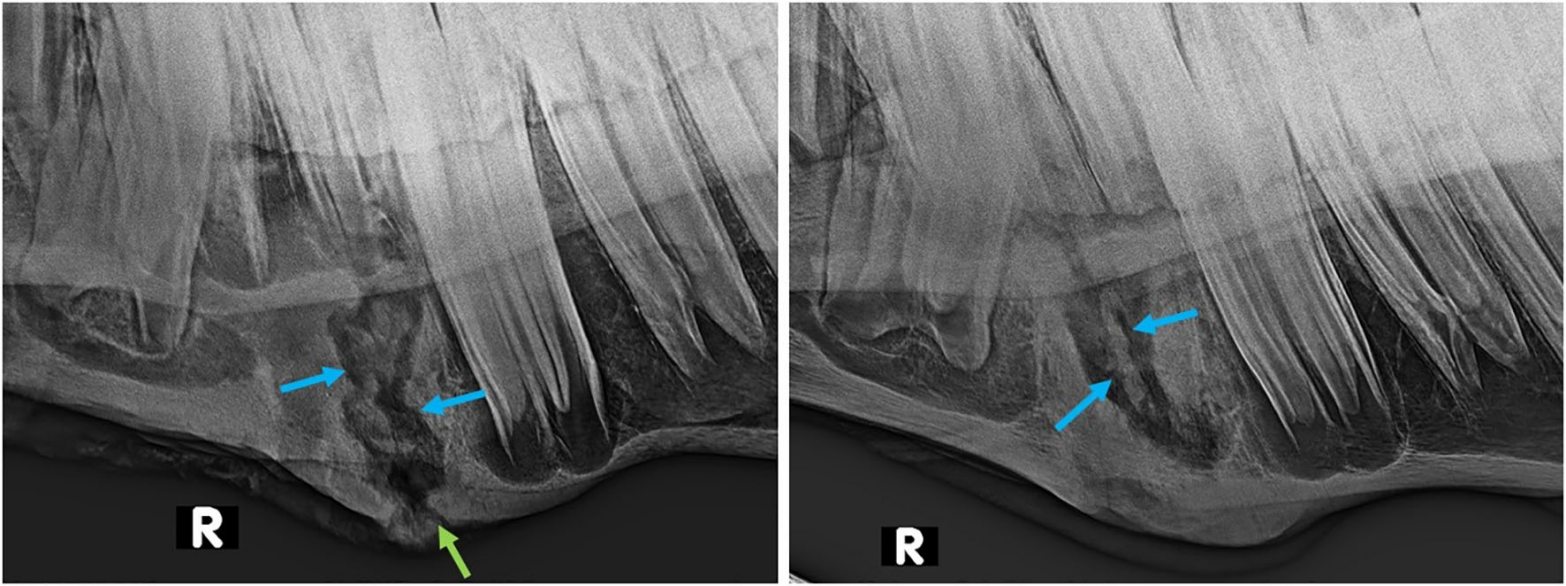
Lateral oblique radiographs of the mandibles of two horses after extraction of 407 s, showing demarcated sequestra (blue arrows) in both. Left (Horse No. 16): External swelling and fistula (green arrow) are still present 10 weeks post-extraction. Right (Horse No. 17): Marked enlargement of the mandibular bone is present 8 weeks post-extraction.

**Figure 8:**
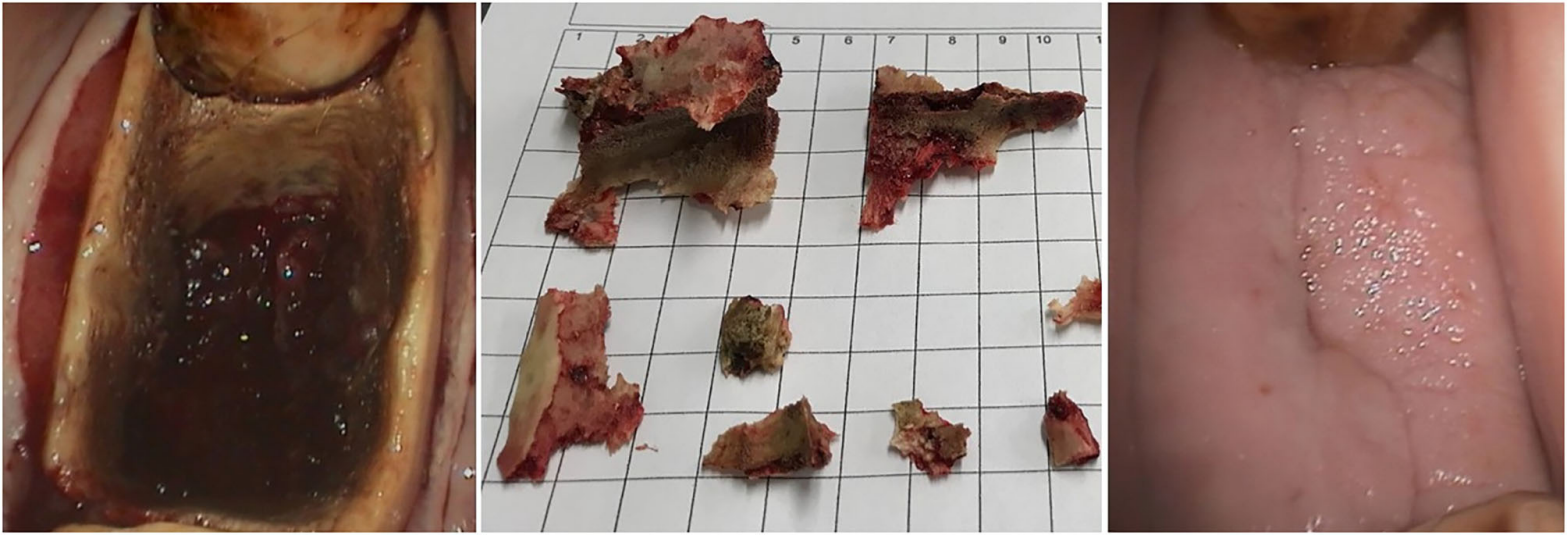
Left: Oral endoscopic view of alveolar sequestration after a 407 extraction (Horse No. 16) by sectioning and repulsion (buccal is to the left). The devitalized bone fragments (middle image) required several treatments for removal. Right: Healing was almost complete after 4 months of additional treatment following exodontia (buccal is to the left).

Sequestration of the alveolus was diagnosed by oral examination and using lateral oblique radiographs in 18/20 cases (Figure 7). The size of alveolar sequestrae varied from small fragments (up to 3 mm wide and <5 mm in length) with some other areas of alveolar healing occurring, to sequestration of the entire alveolus in four horses (Figure 8). Mandibular abscessation and fistula formation varied from fistula a few millimeters wide to extensive skin sloughing (Figure 6, right).

Treatment consisted of repeated sequestrectomy of demarcated loosened alveolar bone fragments, wound debridement, mandibular fistula curettage and non-steroidal anti-inflammatory drugs as required. Systemic antibiotics (trimethoprim sulfadiazine, and metronidazole in some cases) were administered based on microbiological findings in 17/20 cases with clinical discomfort, pyrexia, or evidence of osteomyelitis. Microbiological examination revealed a high prevalence of gram negative obligatory anaerobic bacteria (especially Fusobacterium and Prevotella species) together with *Actinobacillus* and *Streptococcus* species among others. Antibiotic administration was continued until there was an obvious clinical improvement usually for 5–10 days.

If intra-alveolar swabs (impregnated with honey or metronidazole) did not prevent gross alveolar food contamination, a temporary, superficial silicone implant (HS-VPS Hydro Putty Soft® Henry Schein Deutschland GmbH, Hannover, Germany) was used. Once there was an onset of good alveolar healing, horses were discharged from the clinic and thereafter had their progress checked weekly, either by us or the referring veterinarian. Most horses stayed in the hospital for ~5–7 days until discharge, but two were hospitalized for almost 3 weeks at the owners' request. Complete healing was achieved in all cases by a median time of 3 months post-surgery (range 2–5 months). In the more complicated cases that took longest to heal, the costs to treat the complications were significantly higher than the costs for the tooth extraction and perioperative hospitalization itself.

## Discussion

Our clinical impression, that significant post-operative complications associated with mandibular cheek tooth extraction are relatively common has been supported by this study.

Kennedy et al. (3) recently described an increased likelihood of disturbances in alveolar healing following mandibular as compared to maxillary cheek teeth exodontia, especially for mandibular Triadan 06-08 teeth. Apical infections, repulsion technique and horses of younger age were risk factors for post-operative complications (3). From our results, we can draw some similar conclusions.

Teeth extracted because of apical infections that later developed post-operative alveolar sequestration were most prevalent for mandibular Triadan position 07 in young horses (mean 5.5 years) in this study. These findings correlate well with a study by Dixon et al. (5) who found horses with cheek teeth apical infections were a median age of 5 years, with mandibular cheek teeth 07 and 08 most commonly involved. Theories why these teeth positions were so frequently infected soon after eruption include haematogenous infection of enlarged ‘eruption cyst’ that were possibly caused by cheek teeth impaction between adjacent teeth (8) or due to prolonged retention of deciduous cheek teeth remnants. However, in contrast to the study population from Kennedy et al. (3) mandibular Triadan 08 s post-extraction complications were not recorded in our study although similar numbers of Triadan 07 s and 08 s were extracted (55 and 47 teeth, respectively). In contrast to Kennedy et al. (3), this study found the Triadan 09 s to most commonly (7/20) develop post-extraction problems.

Extraction of apically infected mandibular cheek teeth, especially in young horses, is complicated by their very extensive, and largely intact periodontal membranes (some loss at infected apex), that firmly attach their long reserve crowns to the alveoli (6). Increased surgical time, more elaborated extraction techniques, and application of higher forces are often necessary for exodontia in such cases. Extraction, especially elevation, is made more difficult if apical inflammation related deformation of the roots makes this area larger than the remaining alveolus (Figure 3, left). Rarely in chronic cases, these inflammatory processes may even apparently lead to ankyloses of the apex to the mandibular cortex (5), although such dental ankylosis of deciduous teeth, with a number of suggested causes is a relative common condition in children (9).

Appropriate pre-operative radiography is necessary to detect contour changes that could later complicate the extraction procedure and to help plan the most favorable extraction technique. Nonetheless, a standard series of two-dimensional radiographs of the mandible will not fully visualize apical contour changes that expand in a more latero-medial direction. This may increase the risk of underestimating the extent of tooth and alveolar bone deformation. Therefore, computed tomographic imaging should be considered in selected cases, including those with proliferative apical changes that would help with planning the surgical procedure and help to predict the post-operative course.

Following exodontia of mandibular cheek teeth with exostoses in this study, we would recommend that if there is no sufficient progress in loosening the tooth using the standard protocol with elevators and spreaders, pre-operative diagnostic images should be critically reassessed for any findings that could complicate the procedure. For example, in horse no. 16 retrospective examination of the pre-extraction radiographs indicate that oral extraction was likely to fail due to apical enlargement (Figure 3, right). Direct sectioning of this tooth after it was loosened without repeated attempts at elevation may have diminished the extent of post-operative sequestration. We conclude that prolonged oral extraction should not be performed unless some loosening and elevation of the tooth is occurring (independent from surgery time), and an alternative technique should be used to prevent tooth fracture and unnecessary damage to the alveolus and surrounding mandibular bone.

Oral extraction should always be the first method of choice, as several studies have proven the lowest complication rates for this approach (2, 6, 7, 10). However, pre-existing or exodontia-related tooth fractures can make oral extraction impossible (11) and necessitate another extraction method such as minimally invasive lateral buccotomy, intraoral sectioning with a surgical bur or repulsion. Occlusal fissure lines have been frequently identified in equine cheek teeth (12) but to communicate with the pulp in only 23% of cases (13). Such cases may contribute to endodontic diseases and crown fractures as well as to fractures during oral extraction.

We speculate that the more rectangular shaped mandibular cheek tooth as compared to the squarer shaped maxillary cheek tooth makes the former more prone to damage during extraction. Under latero-medial forceps forces, a mandibular cheek tooth cannot “rotate” as well as a maxillary cheek tooth. Not only is a mandibular cheek tooth at higher risk of fracture of the clinical crown with subsequent remaining of the anatomical crown and roots, the extractors may also apply more forces on to the alveolus. The production of bony micro-fractures may cause a diminished blood supply, devitalization, and later sequestration of alveolar bone. Human mandibular bone is a denser structure with a poorer blood supply than maxillary bone (14). If this also applies to horses, it probably further decreases the ability of mandibular bone to recover from exodontia damage.

Following extraction, the alveolar bone is further likely to undergo a diminished blood supply from the damaged or locally absent overlying periodontal membranes. This risk increases if oral extraction is not successful and more invasive exodontia techniques are required.

The most common secondary exodontia technique used in cases where *per os* extraction with forceps was not successful in fully extracting the tooth was intraoral dental sectioning under endoscopic control. Dental sectioning requires good equipment and teamwork, knowledge and experience including a reliable anesthetic protocol. Nonetheless, complications can occur if the horse is not sufficiently chemically restrained or if the alveolus or adjacent soft tissue are damaged directly by the bur or indirectly by conduction of thermal necrosis from the bur. These injuries can damage local blood supply and also provide an entry portal for microorganisms. These areas are also more prone to sequestrate and, if sequestrae are retained in the non-healing alveolus, may predispose to a localized osteomyelitis. Adjunctive water-cooling may offset the risk of thermal necrosis.

Alveolar sequestration was the most common (90%) post-operative complication in this study. Rice and Henry (7) described a sequestration prevalence of 2.4% after oral extractions following partial coronectomy and an overall complication rate of 3.6% (7). Considering that our cohort represents a prevalence of clinically significant sequestration in 18/302 (6%) mandibular cheek teeth in total, the prevalence is 2.5 times higher. Partial coronectomy appears to help loosen the tooth more efficiently and to allow elevation with less force compared to standard oral extraction methods. This may help reduce the prevalence of sequestration. However, the study of Rice and Henry compromised mainly of maxillary cheek teeth extractions. It appears from the current study that maxillary cheek teeth exodontia with subsequent alveolar sequestration is of less clinical concern and will often resolve with minimal effort or even spontaneously as also found by Kennedy et al. (3). Maxillary alveoli have naturally a better draining capacity as wound secretion and small sequestrae will fall out due to gravity. In contrast, empty mandibular alveoli provide a blind ending pocket in which microorganisms and sequesters can remain and promote an ongoing infection in the previously damaged alveolus (3). It is important to remove sequestra in all cases as antibiotic therapy and lavage alone nearly always fails to result in complete resolution of clinical signs and healing of the alveolus (11).

Post-operative complications occurred with all extraction methods in this study. No direct correlation between surgical time, final extraction method and severity of complications was found. One would anticipate a higher complication rate for longer and more complicated dental surgeries. However, one horse (No. 14, an 8 years old Warmblood gelding) where the apically infected 307 was extracted in <30 min, required continued removal of sequestrated bone fragments for over 4 months before complete healing occurred. In contrast, another 8 years old Warmblood gelding from the original cohort of extraction cases, which also had a 307 extracted, via sectioning and repulsion in a surgery that lasted about 4 h, did not develop any post-operative complications. These different responses indicate that the complication rate is not only associated with the extraction method and the course of the surgical procedure, but also with the stage and severity of the underlying disease or a combination thereof. Differences in the host's immune response and in microbial challenge may also influence the development of post-extraction infections (3). Albeit every dental surgery is invariably a contaminated procedure, mandibular bone abscess formation caused by apical infection probably further increases the risk of post-operative sequestration due to high bacterial burdens and focal osteomyelitis. The bacteremia identified during equine dental extractions is not necessarily clinically relevant (15), but it may increase the risk for local complications. Kennedy et al. (3) questioned the efficacy of culturing these sites but suggested that it may be useful in cases where osteomyelitis is identified (3).

The aftercare of the patient and the alveolus likely has an important impact on alveolar healing, although Caramello et al. (2) did not detect any significant association between alveolar packing and delayed alveolar granulation. Currently, there is no objective consensus on the optimal management of equine post-extraction alveoli. Nevertheless, regular alveolar examinations and packing changes, at least weekly post-surgery, are advisable to hopefully reduce microbiological overgrowth and to detect delayed healing at an early stage. Our clinical impression is that alveolar packing with swabs is more effective than silicone implants in cases without fistula formation. Swabs appear to allow for better drainage of secretions and give less resistance to sequestrae separation, leading to improved alveolar healing, but these assumptions need to be proven by clinical studies. There is no objective data on the best type of material to impregnate surgical swabs for alveolar packing. Kennedy et al. (3) used packing with metronidazole and broad-spectrum antibiotics but this did not prevent post-extraction complications in 5.9% of 407 mandibular cheek tooth extractions (3). Therefore, swab impregnation with antibiotics appears to give similar results to the use of medical grade honey (6.6% clinical alveolar post-extraction problems) for the routine packing of alveoli. Considering that antibiotic resistance is increasing, the use of such antibiotic dressings should be limited to cases with a specific indication for antibiotic therapy (16).

Limitations of this study include the incomplete comparison of the outcome for maxillary and mandibular cheek teeth exodontia. A complete evaluation of the post-operative outcome of maxillary cheek teeth extractions would be required to put the general risk factors into perspective. A direct comparison of the underlying dental disease necessitating exodontia, extraction techniques and outcome for all mandibular cheek teeth removals (complications vs. no complications) would further contribute to assessing risk factors for post-extraction complications. Furthermore, due to the retrospective design of this study there was incomplete follow-up information for cases with minor complications resolving spontaneously or treated by the referring veterinarian.

Complications associated with mandibular cheek tooth removal not only escalate the treatment costs, they may also cause more serious morbidity than the initial problem. The treatments to correct these post-extraction problems are often more time-consuming and more difficult than for the original underlying disease. Consequently, it is essential that a thorough clinical and radiographic examination is initially performed in order to decide on the optimal extraction technique. Where the oral extraction process is not proceeding as planned, the radiographs should be reviewed and/ or advanced imaging such as computed tomography sought. Moreover, the risks of such extractions should be disclosed to the owner before the procedure and they should be advised how to recognize post-extraction complications. Increasing our knowledge of possible risk factors for these complications through studies like this and future similar studies will hopefully help to decrease the complication rates.

## Data Availability Statement

The raw data supporting the conclusions of this article will be made available by the authors, without undue reservation.

## Ethics Statement

Ethical review and approval was not required for the animal study because the data used in this study are based on clinical data generated for accountancy and documentation purposes. Our research does not involve any regulated animals, and there were no scientific procedures performed on animals of any kind. For this reason, formal approval by an ethical committee was not necessary under the provisions of the German regulations. Written informed consent for participation was not obtained from the owners because Retrospective analysis of clinical data. No identifiable animal and human data included.

## Author Contributions

All authors listed have made a substantial, direct and intellectual contribution to the work, and approved it for publication.

## Conflict of Interest

The authors declare that the research was conducted in the absence of any commercial or financial relationships that could be construed as a potential conflict of interest.
